# Motor Behavior Changes Are Predictive of Acute Events in Skilled Nursing

**DOI:** 10.1093/geroni/igab046.155

**Published:** 2021-12-17

**Authors:** Mary (Libbey) Bowen, Meredeth Rowe, Pamela Cacchione, Ming Ji

**Affiliations:** 1 University of Delaware School of Nursing & Cpl. Michael J. Crescenz VA Medical Center, Newark, Delaware, United States; 2 University of South Florida, Tampa, Florida, United States; 3 University of Pennsylvania, University of Pennsylvania School of Nursing, Pennsylvania, United States

## Abstract

Background: Common acute medical conditions among older adults with dementia in skilled nursing include falls, delirium, and pneumonia. This study utilized a sensor technology to examine how motor behaviors may predict these acute events. Methods: Radio frequency identification (RFID) technology continuously measured time and distance travelled, gait speed, and continuous walking with little/no breaks (paths) across 3 long-term facilities for up to 1 year (N=51). Change point analysis estimates the probability of whether a sudden change occurred and provides the location of the change point (in days prior to the event) in a time series model. Results: Gait speed had very low probability to detect a change point across all events (22 falls, 10 delirium and 8 pneumonia). Sensitivity estimates ranged from 63% (number of paths) to 90% (distance travelled) for a fall; 37.5% (number of paths) to 100% (rest of the motor behaviors) for pneumonia. Except for gait speed, all other motor behaviors had high probability (100%) to detect a delirium change point. There was intra-individual variability in the location of the change points (mean of 10 days). Linear regression models for time and distance travelled using baseline predictors of age, ethnicity, gait and balance explained 89% and 90% of the variance in change point locations. Conclusions: Prior to an acute event there is a significant change in motor behavior, suggesting these are an early signal that may be used to prevent a fall or provide for the earlier recognition and treatment of delirium and pneumonia.

